# Posttranscriptional Expression Regulation: What Determines Translation Rates?

**DOI:** 10.1371/journal.pcbi.0030057

**Published:** 2007-03-23

**Authors:** Regina Brockmann, Andreas Beyer, Jürgen J Heinisch, Thomas Wilhelm

**Affiliations:** 1 Theoretical Systems Biology, Leibniz Institute for Age Research, Fritz Lipmann Institute, Jena, Germany; 2 Fachbereich Biologie/Chemie, AG Genetik, Universität Osnabrück, Osnabrück, Germany; 3 Department of Bioengineering, University of California San Diego, La Jolla, California, United States of America; University of Texas, United States of America

## Abstract

Recent analyses indicate that differences in protein concentrations are only 20%–40% attributable to variable mRNA levels, underlining the importance of posttranscriptional regulation. Generally, protein concentrations depend on the translation rate (which is proportional to the translational activity, TA) and the degradation rate. By integrating 12 publicly available large-scale datasets and additional database information of the yeast *Saccharomyces cerevisiae,* we systematically analyzed five factors contributing to TA: mRNA concentration, ribosome density, ribosome occupancy, the codon adaptation index, and a newly developed “tRNA adaptation index.” Our analysis of the functional relationship between the TA and measured protein concentrations suggests that the TA follows Michaelis–Menten kinetics. The calculated TA, together with measured protein concentrations, allowed us to estimate degradation rates for 4,125 proteins under standard conditions. A significant correlation to recently published degradation rates supports our approach. Moreover, based on a newly developed scoring system, we identified and analyzed genes subjected to the posttranscriptional regulation mechanism, translation on demand. Next we applied these findings to publicly available data of protein and mRNA concentrations under four stress conditions. The integration of these measurements allowed us to compare the condition-specific responses at the posttranscriptional level. Our analysis of all 62 proteins that have been measured under all four conditions revealed proteins with very specific posttranscriptional stress response, in contrast to more generic responders, which were nonspecifically regulated under several conditions. The concept of specific and generic responders is known for transcriptional regulation. Here we show that it also holds true at the posttranscriptional level.

## Author Summary

Large-scale mRNA concentration measurements are a hallmark of our post-genomic era. Usually they are taken as a surrogate for the corresponding protein concentrations. For most genes, proteins are the actual cellular players, but up to now it has been much more difficult to measure protein concentrations than mRNA concentrations. However, due to numerous posttranscriptional regulation mechanisms, mRNA levels only partly correlate with protein concentrations. Based on thoroughly composed reference datasets for protein and mRNA concentrations in yeast under standard growth conditions, we report the best corresponding correlation so far. We took into account additional factors, beyond mRNA concentrations, that influence protein levels in order to improve protein level predictions. Extending our previous approach, where ribosome occupancy and ribosome density were considered, we now also consider ORF-specific translation elongation rates. Different measures for elongation velocity were examined, and the codon adaptation index was found to be most appropriate. Moreover, saturation kinetics were introduced to better describe the translation process. The general findings were also applied to four stress conditions. Three new concepts, translation on demand, just-in-time translation, and general and specific posttranscriptional stress responders, are discussed.

## Introduction

Although mRNA concentrations are widely used as a surrogate for protein abundances, studies comparing mRNA and protein expression on a global scale indicate that mRNA levels only partly correlate with the corresponding protein concentrations [1–12]. It has been estimated that protein concentrations are determined by the corresponding mRNA concentrations by only 20%–40% [11,13]. Thus, for a better interpretation of results obtained from mRNA measurements, a deeper understanding of translational regulation is urgently required [14–16].

To study the fundamental role of posttranscriptional regulation, we focused on S. cerevisiae as one of the most thoroughly investigated model organisms, where mRNA concentrations and even protein concentrations are available for most genes. More specifically, we were interested in (i) identifying the most important factors regulating translation rates and (ii) specific translational regulation under different conditions. The translation rate is proportional to the translational activity (TA) [9,10,17], which we previously calculated as the product of mRNA abundance, ribosome occupancy, and ribosome density [10]. Ribosome occupancy (*ribocc*) is the fraction of mRNA molecules with at least one ribosome, and the ribosome density (*ribden*) is the number of ribosomes on active mRNAs divided by the transcript length [18]. Hence, *ribden* takes into account that longer transcripts take longer to be translated, and require a larger number of bound ribosomes to achieve the same synthesis rate (number of new proteins per time).

Here, we additionally account for ORF–specific translation elongation velocity. It depends on the amino acid composition of the corresponding protein and the availability of the needed tRNAs. We discuss different measures for the elongation velocity based on tRNA concentrations. Interestingly, we found that the codon adaptation index (CAI) was the best measure for the speed of translation elongation, because it improved the correlation between TA and protein concentrations more than any of the other measures tested. The CAI was initially introduced as a measure for selection of optimal codons in ORFs based on highly abundant mRNAs [19]. It is defined as the geometric mean of the relative synonymous codon usage (RSCU) values for all codons of a given ORF, normalized by the maximum possible mean RSCU value (the RCSU value for a codon is the observed codon frequency divided by the expected frequency for equal codon usage). The CAI has also been used to predict protein concentrations [1,2].

Whereas up to now linear kinetics have been assumed for the TA [8,10,20], here we demonstrate that accounting for nonlinear saturation improves the protein concentration prediction: the TA–protein correlation is improved assuming Michaelis–Menten kinetics for the three factors influencing the translation initiation. Using our newly calculated TAs and our newly composed reference dataset for protein concentrations, we were able to deduce degradation rates for 4,125 proteins. Comparison of our predicted values with measured protein half-lives [21] shows that including the CAI and accounting for saturation significantly improved our predictions.

Previous studies on posttranscriptional regulation that included protein concentration measurements in yeast focused either on standard conditions [1,5,8,10] or dealt with just one stress condition [3,4,6,9]. Other studies only measured *ribden* changes without considering the respective protein concentration changes [22]. Here, we present the first comprehensive analysis of different stress conditions by combining existing experimental data. For this purpose we used all four published large-scale datasets that tested the relative change of both mRNA and protein abundances upon exposure to different stress conditions [3,4,6,9]; two other studies were published after completion of this work and could therefore not be analyzed in detail here [15,16].

Our analyses of the stress data support the finding that considering saturation kinetics improves the quantification of posttranscriptional regulation. We also confirmed the previously introduced concept of translation on demand, concerning proteins that are quickly needed in response to a (stress) stimulus [10]. In such situations the usual order of events, with transcription and subsequent translation, may be too slow for an appropriate physiological reaction. Instead, the cell might keep a constant level of reservoir mRNA, which is blocked for translation. Processing bodies (P-bodies) may play a role in mRNA storage. It has been shown that processing bodies accumulate mRNAs for subsequent degradation [23], but they may also store mRNA for later translation [24]. Translation initiation might also be blocked by 5′ binding proteins or alternative 5′ leaders [25]. If the corresponding protein is rapidly needed (for instance, after cell exposure to perilous conditions), the cell can then immediately start with translation. The detailed analysis of 62 proteins that were measured under all four conditions confirmed the existence of translation on demand and identified additional candidates. Another means for fast stress response is the continuous synthesis and destruction of proteins under normal conditions. Upon stress the protein turnover can be stopped to quickly elevate protein concentration [26]. Since this mechanism does not change the translation rate, it is not considered to be translation on demand.

Based on the available experimental data, it is possible to separate proteins with distinct posttranscriptional regulation under one specific condition from others that are regulated in a more generic way. This extends the previous notion of generic and specific stress response from the level of transcriptional to posttranscriptional expression regulation [27].

## Results

Our first goal was to establish a reliable set of experimental data to investigate the correlation between mRNA abundance and observed protein concentrations. We integrated various published datasets for S. cerevisiae [1,2,7,28] and obtained protein and mRNA concentrations under standard conditions for 4,152 ORFs, representing the largest corresponding dataset so far. Correlations between protein concentrations and other properties were computed using the Spearman rank correlation coefficient *r_s_*. The *r_s_* was preferred over the Pearson correlation coefficient, because the former makes no assumptions about the underlying distributions of the variables. It has previously been shown to perform better for the analysis of this kind of data [2]. The global *r_s_* between mRNA and protein abundance was 0.63, which is the best large-scale correlation reported for yeast so far [8,10,16]. This high correlation also underlines the quality of the integrated dataset. After completing the computational part of this study, another large-scale protein concentration dataset was published [15]. This new dataset could not be included in this study. However, we computed the correlation of our integrated dataset to those independent measurements. Our integrated protein concentrations were more strongly correlated to those measurements (*r_s_* = 0.65) than any of the individual input datasets alone (*r_s_* ranging from 0.39 to 0.62). We thus conclude that our protein dataset is comparably robust and that our conclusions are unlikely to change significantly if the new data were included. However, the remaining deviations between those measurements reenforce the finding of Newmann and co-authors that biological noise in protein concentrations can be considerable [15].

### Factors Influencing Translational Activity

**Figure 1:**
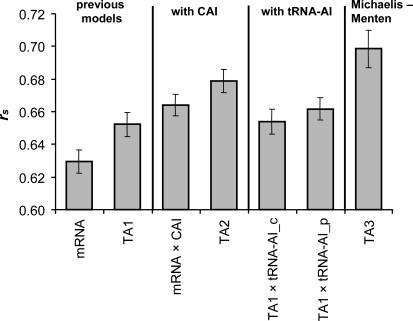
Correlation of Protein Abundance and Different Translation Rates Shows Spearman rank correlation (*r_s_*) of TA versus reference protein abundance; error bars indicate ± one standard deviation based on random subsamples. *TA*1 = mRNA × *ribocc* × *ribden*; *TA*2 = *TA*1 × CAI; *TA*3 assumes Michaelis–Menten kinetics for the TA (Equation 3). The tRNA–AIs were calculated as described in Methods; tRNA–AI_p indicates the codon–tRNA assignment according to [42] and tRNA–AI _c the assignment according to Crick's wobble rules [43]. All correlations are based on 4,123 ORFs. Accounting for tRNA–AI_p slightly improves correlations compared with *TA1* alone. However, *TA2* (with CAI) performs better. Overall, considering saturation, (*TA3*) gave the best results.

We quantified the relative importance of various factors for the determination of TAs (Figure 1). Considering additional factors always improved the correlation compared with using mRNA levels alone (*r_s_* increases from 0.63 to 0.70; Figure 1). In addition to mRNA concentration, *ribocc,* and *ribden,* we also took into account sequence-based aspects of elongation efficiency. In Escherichia coli, translation of major codons occurs 3-fold to 6-fold faster [29,30] and 10-fold more accurately [31] than translation of minor codons, which also reduces the cost of GTP-dependent proofreading [32]. We thus used the CAI as an indirect measure of elongation velocity, because it is easily accessible for all yeast ORFs. Figure 1 shows that accounting for the CAI further improved the correlation to protein concentrations (*r_s_* = 0.68). Most of the factors contributing to TA are correlated with each other, e.g., CAI correlates with mRNA levels. To demonstrate that each factor independently contributes to the TA, we computed partial correlation coefficients for all relevant combinations of factors (Table 1). This analysis shows that (i) every factor independently carries significant information about the translation rates and (ii) the CAI and the ribosome-related factors (*ribocc* × *ribden*) contributed about equally to the overall TA. Further, we determined the significance of regression improvements by randomly subsampling the datasets. Figure 1 shows the standard error of the regression coefficients obtained by randomly subsampling the datasets (two-thirds of the proteins were randomly sampled). To determine if the regression improvements were caused by only few proteins (outliers), we checked if the improvements also hold for the subsamples (see Methods). We tested 1,000 random subsamples and always observed relative improvements when considering more factors for TA prediction.

**Table 1:**
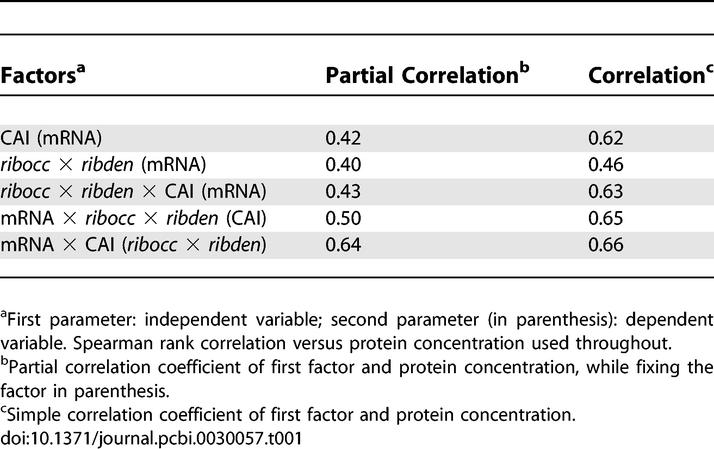
Contribution of Each Factor to the Translational Activity

### Influence of the tRNA Adaptation Index

The CAI is exclusively based on the frequency of codons in highly expressed genes. Thus, it only indirectly accounts for tRNA availability. A direct quantification of tRNA concentrations for the respective codons might give an improved descriptor for an ORF's tuning for fast elongation. To directly account for tRNA availability, we introduce the tRNA adaptation index (tRNA–AI) ([33]; for calculation, see Methods). However, problems for the calculation of tRNA–AIs are the lack of measured tRNA concentrations and the ambiguous assignment of the 42 yeast tRNAs to the 64 codons. We tested two different tRNA–AIs (see Methods). The correlations to the CAI are *r_s_* = 0.91 for tRNA–AI_p and *r_s_* = 0.55 for the tRNA–AI_c. Next, we replaced the CAI in *TA2* (Equation 2) by either of the tRNA–AIs to test if the tRNA–AIs are equally or more predictive than the CAI. Figure 1 shows that the strict assignment (tRNA–AI_p) yielded a protein–TA correlation slightly better than the correlation with *TA1* (*r_s_* = 0.66 versus 0.65), whereas using tRNA–AI_c yielded no improvement compared with *TA1* (*r_s_* = 0.65). Hence, accounting for tRNA–AI_p improved the predicted translational activity, while tRNA–AI_c was not predictive for TA. Thus, (i) the tRNA concentration is indeed an important factor for translation, and (ii) the speed of elongation seems to be mainly determined by the availability of tRNAs with perfectly matching anticodons. This observation was supported by the findings of Rocha [34], who showed that the perfect match model is more likely to mimic in vivo conditions than the frequency model. Yet, the protein–TA correlation based on the CAI was still better than the one based on the tRNA–AI_p. This suggests that the CAI contains information beyond the tRNA gene copy number. For instance, the codon–anticodon interaction strength might also affect the efficiency of elongation [34,35]. Therefore, all following estimations of TA are based on CAI, and the tRNA–AIs were not used any further.

### Saturation Effects in the Translation Rate and Calculation of Protein Half-Lives

**Figure 2:**
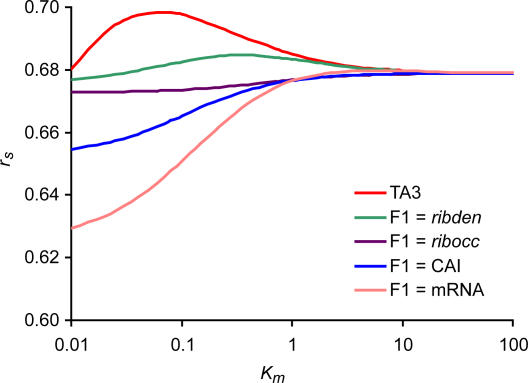
Saturation of the Transactional Activity Shows Spearman rank correlation (*r_s_*) of different models for TA prediction as a function of the Michaelis constant, *K_m_*. All four factors (F) contributing to TA (mRNA, *ribocc, ribden,* CAI) were tested for saturation individually and in combination. The different colors indicate the models for TA prediction:

So far we assumed a linear relationship between the TA and the factors defining it. However, because energy, as well as the numbers of tRNAs, amino acids, and ribosomes available in a cell, are limited, actual TA may saturate for high mRNA concentrations. To test this hypothesis, we computed TAs using different possible kinetic relationships, which were all based on Michaelis–Menten kinetics (Figure 2). Ribosome density was the only single factor that showed a slight saturation effect. However, by combining mRNA concentration, *ribocc,* and *ribden* in one saturation term (named *TA3*, see Equation 3), we significantly enhanced the protein–TA correlation (see Methods and Figure 2). This combined term quantifies the number of initiation events for the given ORF.

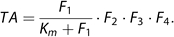 The product of mRNA × *ribocc* × *ribden* in the saturation term (i.e., TA3) yielded the best correlation with reference protein concentrations. The value without any saturation (*r_s_* = 0.68) is approached for *K_m_* → ∞.

Several lines of evidence indicate that the small improvement obtained by accounting for saturation is of true biological relevance: first, we wanted to know if the saturation may be biased by a specific experimental technique. To test for such bias, we divided the dataset into a training set and a test set based on different measurement techniques (Figure S1 in Protocol S1). This analysis showed that, even if we train the Michaelis constant, *K_m_,* on protein concentrations obtained with one experimental method, it also improved the correlation for protein concentrations from other experimental methods. Thus, the type of kinetics suggested by Equation 3 is independent of the experimental techniques employed. Second, we randomly split the data into training (two-thirds) and test (one-third) sets. We determined *K_m_* based on the training data and applied it to the test data. We repeated this test 50 times, and the resulting correlations of *TA3* in the test data were consistently high (Table 2). The *K_m_* values determined for *TA3* were remarkably stable (coefficient of variation *CV* = 0.18, Table 2). As opposed to that, the *K_m_* values for the alternative models were more sensitive, which additionally supports that our model better fits the observed data. Finally, we analyzed the respective correlations for different functional groups of proteins (Figure S2 in Protocol S1). We observed at least a slight improvement when using *TA3* for 17 out of 18 functional groups. The final TAs predicted for 6,063 ORFs, as well as respective protein half-life descriptors (PHD, see Methods) for 4,125 ORFs, are provided in Table S1. The PHD is only a relative descriptor of protein half-life [10], but we expect that the predicted PHDs correlate with measured protein half-lives. We compared our PHDs with recently published protein half-lives for yeast [21]. PHDs determined with *TA1* or *TA2* were not significantly correlated with the measured protein half-lives (|*r_s_*| < 0.1, p > 0.01), whereas PHDs based on *TA3* exhibited a weak but significant correlation (*r_s_* = 0.24, *p* < 10^−40^). The predicted PHDs are based on five values, most of which are noisy (except for CAI); the measured protein half-lives are also subject to biological noise. Nevertheless, the above significant correlation provides confidence that protein stability prediction via PHD is possible—at least within certain limits. We expect that future PHDs will improve further as more and more precise data become available.

**Table 2:**
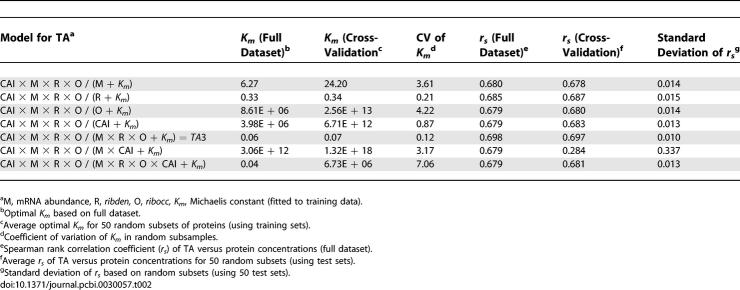
Comparison of Different Saturation Models (Cross-Validation)

### Translation on Demand

To systematically identify candidates for translation on demand, we developed an integrated score. Translation on demand is indicated under normal conditions by (i) a small number of ribosomes translating a given ORF, and (ii) by a small number of proteins produced per transcript. Thus, *ribocc* × *ribden* serves as a first indicator, and the ratio of proteins per mRNA (PRR) is used as the second predictor. A low value for both, *ribocc* × *ribden* and PRR, indicates strong evidence for translation on demand. Next, we computed an integrated score to rank genes with respect to their potential for translation on demand on a single scale. This score is defined as the weighted sum of *ribocc* × *ribden* and PRR (both normalized by the median values). A low value of this score is indicative for translation on demand. We also tested other scores, but they performed significantly worse than this scoring scheme. For instance, the (weighted) product of *ribocc* × *ribden* and PRR may yield low scores even if one of the two descriptors is high. This is particularly problematic for proteins with short half-lives, because they might exhibit low PRRs without actually being translated on demand.

Due to the necessity for relatively fast responses, components of signal transduction chains are probable candidates for regulation at the translational level (in addition to covalent modifications governing their activities). To test this hypothesis, we assessed the overlap of the top 100 and the top 500 genes according to our score with gene categories from the Munich Information Center for Protein Sequences (MIPS) (http://mips.gsf.de) and the Saccharomyces Genome Database (SGD) (http://www.yeastgenome.org). As shown in Tables S3 and S4, these top-scoring candidates were indeed significantly enriched for genes with functions related to signal transduction and, interestingly, to transcriptional regulation. This suggests that transcription factors are among the first proteins that are rapidly synthesized upon detection of stress signals. We also analyzed the correlation of protein and mRNA concentrations within groups of genes with similar functions (functional modules [10]). Figure S2 in Protocol S1 shows that modules related to signal transduction (i.e., those likely to contain many proteins that are subject to translation on demand) exhibit particularly weak protein–mRNA correlations. However, after accounting for posttranscriptional regulation, those correlations improved. Hence, the protein–mRNA correlation in regulatory modules gets distorted by posttranscriptional processes.

### Posttranscriptional Regulation under Stress Conditions

Next we asked whether our results, obtained under standard conditions, also hold under stress conditions. We considered all four available datasets with measured mRNA and protein concentrations under standard and stress conditions (see Methods). Unfortunately, most studies did not provide ribosome densities and occupancies, so we only considered changes in protein and mRNA concentrations under stress.

Both mRNA and protein concentration changes were measured for 1,216 ORFs in response to at least one of the four stimuli. When a given ORF is translated on demand under one of the tested conditions, one expects to see a greater change in its protein concentration than in its mRNA level. Therefore, the ratio of protein to mRNA concentration changes was computed for all available genes and conditions. These ratios served as additional evidence for translation on demand, and may help identify targets for future research. Accordingly, we took a closer look at high-ranking genes (rank < 500) exhibiting strongly elevated protein concentration changes under at least one of the four tested stress conditions. Concentrations of 20 proteins were elevated at least two times more than their corresponding mRNA (Table S5). It would be most interesting to analyze the posttranscriptional regulation of these genes more in detail in the light of these results.

**Figure 3:**
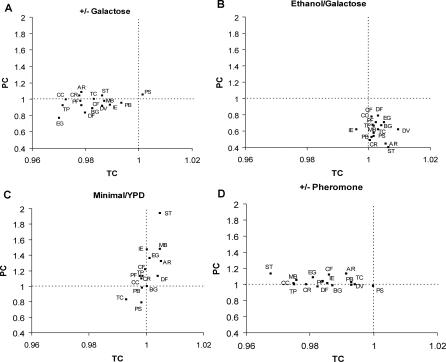
Change of Translation Rate and Protein Change in Response to Different Stimuli Median protein and translation rate ratios are shown for the conditions ± galactose (A), ethanol/galactose (B), minimal medium/normal medium (C), ± mating pheromone (D). Translation rates were calculated according to Equation 3, assuming constant *ribden* and *ribocc* (because those changes were unavailable for all but one condition). Proteins were functionally grouped based on MIPS annotation. AR, protein activity regulation; BG, biogenesis; CC, cell cycle/DNA processing; CF, cell fate; CR, cell rescue/defense/virulence; DF, differentiation; IE, interaction with cellular environment; MB, metabolism; PB, protein with binding function; PF, protein fate; ST, cellular communication/signal transduction; TC, transcription; TP, cellular transport.

Figure 3 shows the protein change (PC) and change of translation rate (TC) for different stimuli and functional groups. Viewing the data this way emphasizes the high specificity of transcriptional and posttranscriptional responses under different conditions. Whereas a lack of amino acids (Figure 3C) induces dramatic posttranscriptional changes, the mating pheromone induces stronger transcriptional changes (Figure 3D). The figure also highlights the functional difference in protein concentration regulation. For instance, proteins involved in signal transduction (group ST) are clearly strongly upregulated at the posttranscriptional level in the minimal medium. This once more underlines the importance of fast posttranscriptional changes of protein concentrations (e.g., translation on demand) especially for signaling proteins. Proteins involved in protein synthesis, on the other hand (group PS), were downregulated, presumably because the overall protein synthesis was reduced in minimal media. When switching the energy source (Figure 3A), changes in protein synthesis were less drastic, while energy-related proteins (group EG) changed most at both the transcriptional and posttranscriptional levels.

The experimental datasets contain 62 ORFs with mRNA and protein ratios measured under all four stress conditions. By combining these data, we were able to analyze the differential regulation of these proteins under several conditions. One noteworthy candidate for translation on demand is YPL028w (also known as ERG10, LPB3, or TSM0115), which codes for an acetyl-CoA C-acetyltransferase. This enzyme is involved in the first step of mevalonate biosynthesis [36]. Accordingly, it was substantially upregulated in the galactose experiment (6.7-fold change) without significantly changing its mRNA concentration (0.7-fold change). Another protein catalyzing the first step of a pathway (serine and glycine biosynthesis) is Ser3p [37]. Although the mRNA concentration was also upregulated (4.2-fold change) upon exposure to minimal medium [4], the increase in protein concentration was much higher (18.2-fold change). Whereas it is well-established that genes are transcribed in the order of the appearance of their products in metabolic pathways (just-in-time transcription [38,39]), YPL028w and Ser3p represent the first hints for a similar regulation at the translational level (just-in-time translation). Thus, enzymes catalyzing important first steps in metabolic pathways might also be regulated at the translational and post-translational level (e.g., allosteric control or covalent modifications) to ensure a fast cellular response.

Table 3 shows the PC/TC ratio for selected proteins. The complete table, with all 62 proteins measured under all four conditions, is shown in Table S6. On the top of the table are all proteins whose maximum PC/TC is at least 2.5 times higher than for any of the other conditions—these are specific posttranscriptional responders. At the bottom of Table 3 are general responders, i.e., proteins without a distinct posttranscriptional upregulation under one specific condition. Hence, there are two classes of proteins: the first class contains proteins that are strongly posttranscriptionally upregulated under just one of the four tested conditions. These proteins are likely to be stress specific. The second class contains proteins that are either not distinctly regulated at the posttranscriptional level (i.e., the protein and mRNA changes were similar) or that are upregulated under several stress conditions. We found that proteins involved in amino acid metabolism or protein fate exhibit condition-specific posttranscriptional regulation, in contrast to proteins with more generic cellular functions (those at the bottom of Table 3, e.g., proteins involved in translation). Importantly, this pattern only emerged after accounting for the saturation of translational activity (Equation 3, Table S6).

**Table 3:**
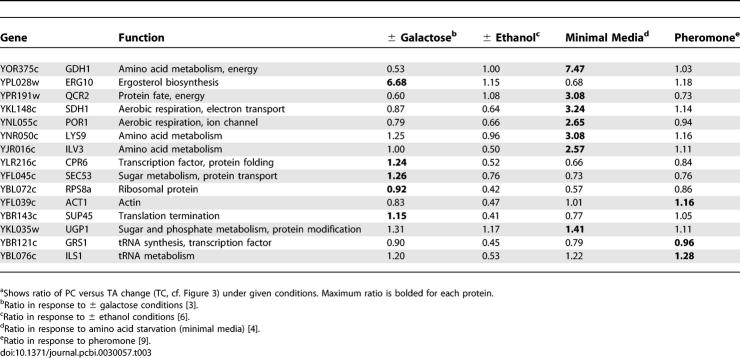
Selected Examples of Proteins with Clear Patterns of Specific Stress Response (First Seven Rows) or Generic Stress Response (Last Eight Rows) after Accounting for Saturation of the Translational Activity^a^

## Discussion

It is increasingly recognized that mRNA abundances are only a weak surrogate for the corresponding protein concentrations [13], and it has been proposed that posttranscriptional control of gene expression is at least as important as the better-studied transcriptional regulation [14]. Our work contributes to a better understanding of posttranscriptional regulation by taking into account as much information as possible in addition to mRNA concentrations. In our previous work [10], we did not consider any data describing the translation elongation velocity. Here we introduced the CAI as an additional factor and we demonstrated that it is currently the best corresponding measure. A systematic analysis of all TA factors reveals that the CAI contributes additional independent information for understanding translation rates, and it is at least as important as *ribocc* and *ribden* together. Moreover, for the first time we tackled the problem of the functional relationship between the TA and the contributing factors. The proposed Michaelis–Menten kinetics implies that the concentrations of highly abundant mRNAs have to change much more drastically to achieve a significant change of protein concentrations. The transcripts of many signalling proteins, like transcription factors, are often expressed at comparably low concentrations, facilitating sensitive and significant changes in response to stress. These findings are also in line with the previous observation that protein concentrations tend to be less noisy if transcript levels are high [20]. We found that only the initiation-related factors (mRNA concentration, *ribocc,* and *ribden*) were subject to saturation, whereas the CAI contributed linearly to TA. In other words, once translation started, it progressed as quickly as permitted by the sequence [18]. Importantly, our results imply a protein-specific saturation, as opposed to a global reduction in translation (e.g., due to energy exhaustion). First, each protein requires a distinct set of amino acids, and hence also a distinct set of tRNAs, for its synthesis. Hence, these resources could be exhausted if certain mRNAs were excessively translated. Second, translation is often conducted in a site-specific manner, i.e., transcripts are transported to specific cytoplasmic sites where the protein products are needed [40,41]. Excessive translation can therefore exhaust resources in those cellular regions, whereas translation may remain unaffected at other sites. In this context it is also important to remember that log-growth conditions are not the normal environmental conditions to which yeast cells have been adapted. Natural conditions are much more characterized by nutrient limitations. Hence, it is likely that several proteins are synthesised beyond their optimal limits in fast-growing cells under ideal lab conditions.

It is well-established that the input data used for this study are noisy [5,8,10]. General conclusions would be affected by a systematic bias caused by the noise in the data. Newman et al. [15] found a correlation between protein abundance and biological variability. However, such bias does not affect average concentrations for populations of cells. In fact, Newman et al. report a good correlation of their protein concentrations with previous population-based measurements. It should be noted that our main results are robust to noise in the data: an additional ORF-specific factor accounting for the speed of translation always improves the TA–protein correlation, regardless of whether we use the tRNA–AI or the CAI (Figure 1). Also, there is an improvement of TA–protein correlations by using saturation kinetics for a range of about two orders of magnitude for the *K_m_* value (Figure 2).

Based on our newly calculated TAs, we propose degradation rates for 4,125 proteins. Comparison with the recently published study of the first large-scale measurement of protein turnover [21] reveals that our calculation outperformed previous approaches [10]. Deviations between our predicted values and the measurements were partly due to noise in the data, but they might also pinpoint potential additional posttranscriptional control steps, which should trigger more detailed investigations of these ORFs.

The consideration of all available large-scale data on stress response in S. cerevisiae enabled us to confirm the previously introduced concept of translation on demand [10]. Additionally, based on the analysis of all 62 genes with measured protein concentrations under all four conditions, we demonstrated the first evidence to our knowledge for generic and specific posttranscriptional stress responders. Several proteins that were posttranscriptionally upregulated under only one of the tested conditions might, of course, also respond under other, yet-untested conditions. However, the distinct patterns that already emerged based on the available data indicate that cells use similar regulatory schemes of generic and specific responses to tackle threats at the transcriptional and posttranscriptional level. Many of the translation-on-demand candidates did not show any significant upregulation under any of the tested conditions. The majority of them were not even measured under all four conditions. Also, these conditions only represented a small subset of the possible threats that yeast has adapted to. Clearly, the investigation of the posttranscriptional stress response is lagging behind the corresponding analysis at the transcriptional level. By combining all available information, it might be possible to nail down those conditions under which the translation-on-demand candidates respond at the posttranscriptional level and to experimentally verify the predictions. The in silico analyses presented here will help to streamline those experimental efforts.

## Materials and Methods

### Data used.

A complete list of all data used is presented in Table S1. Only genes occurring in MIPS and/or SGD were considered. The mRNA concentrations for standard conditions were taken from our previous work [10], which were derived from a pool of 36 independent mRNA abundance measurements from different research groups. Protein concentrations for standard conditions of four measurements [1,2,7,28] were normalized by nonlinear regression, and the median was taken as the reference value for each ORF (Table S1). The following equations were used to map the reported measurement value (*meas*) onto a common scale:

Ghaemmaghami et al. [7]: selected value = original data

Prot Futcher et al. [2] [10^3^ copies/cell]: selected value = 9,710.7 · 

*meas* ^0.4293^

Prot Gygi et al*.* [1] [10^3^ copies/cell]: selected value = 10,977 · 

*meas* ^0.2662^

Prot Liu et al. [28] [relative abundance]: selected value = 2,108.2 · 

*meas* ^0.516^

The four stress datasets were taken from [3] (shift from glucose to galactose), [6] (ethanol), [4] (minimal medium), and [9] (exposure to pheromone). Genes were grouped according to the functional protein classification in MIPS, http://mips.gsf.de/genre/proj/yeast/, where one gene can be assigned to several groups.

### Translational activities and protein half-life descriptor.

The TA is a measure for the ORF-specific translation rate. The true translation rate is the product *k_p_* × *TA* [10], where we assume an ORF-independent rate constant, *k_p_*. We tested different variants of estimating the TA from measured data. The first (*TA*1) has been suggested previously [9,10]:

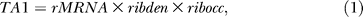
where *mRNA* is the mRNA concentration of that gene [9,18]. Next, we included the CAI:

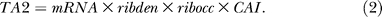



Finally, we assumed Michaelis–Menten kinetics:

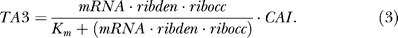



The *K_m_* for the Michaelis–Menten kinetics (*K_m_* = 0.06) was determined by maximizing the correlation between *TA*3 and protein abundance (Figure 2).

The protein half-life descriptor, PHD, was calculated according to [10], but using *TA*3 as an improved measure of translational activity:

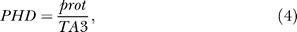
where *prot* is the reference protein concentration of the corresponding gene (see the section Data used).

When comparing the predicted PHDs with measured protein degradation rates from [21], we used two different datasets: the first set contained all proteins with the half-lives as reported by [21], and the second set contained only proteins with half-lives shorter than 300 min. The second dataset was used because short half-lives are more reliable [21]. Correlations with both datasets were very similar, especially the significant correlations (*r_s_* = 0.2427 and 0.2425, respectively).

### Correlation of protein concentrations with mRNA abundance and TA.

Correlations were quantified using the Spearman rank correlation coefficient, which is in agreement with previous studies [5,10]. All reported correlations were based on at least ten data points. Although some of the correlations in Figure 1 can be computed for more proteins than others, all correlations reported were performed on the same set of proteins to avoid biases due to different sample sizes.

### Significance of correlations.

All correlations reported in Figure 1 were significantly different from zero (*p* < 10^−16^). Variability of the correlation coefficients was tested by randomly subsampling two-thirds of the proteins. The *r_s_* was computed for each subsample, and its standard error was computed (Figure 1). To test the significance of correlation improvements, the correlations (r_s_|mRNA, r_s_|TA1, r_s_|TA2, r_s_|TA3) were compared for each subsample individually. In all 1,000 subsamples, *r_s_*|*TA1* > *r_s_*|mRNA, *r_s_*|*TA2* > *r_s_*|*TA1,* and *r_s_*|*TA3* > *r_s_*|*TA2*. This test demonstrated that the respective improvements were not dependent on some specific outliers. We also tested for the significance of individual factors by computing partial correlation coefficients (see main text and Table 1).

### Calculation of the tRNA adaptation index.

The definition of the tRNA–AI is similar to the CAI [19], whereby the RSCU value was replaced by the gene copy number (GCN) of the corresponding tRNA. Under normal growth conditions, the GCN can be used as a measure of tRNA concentrations [42]. The relative adaptation value *w_k_* of a codon *k* is the GCN of that tRNA compared with the maximal GCN for that amino acid:

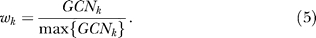



Two assignments of tRNAs to codons were tested. According to [42], each codon gets assigned only one tRNA (the perfectly matching tRNA according to Watson–Crick base pairing, *w_p*). The wobble rule introduced by Crick [43] assumes that some codons can be recognized by several tRNAs. In the corresponding second model, the GCNs were added up (*w_c*) [33].

In accordance with the definition of the CAI, we defined the tRNA–AI of a gene with *L* amino acids as the geometric mean of *w_k_*:

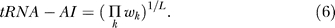



## Supporting Information

Protocol S1 Additional Results (402 KB DOC)

Table S1 All Considered Original Experimental Data and Deduced Calculated Data (2753 KB XLS)

Table S2 Correlation of Measured and Calculated Protein Half-Lives (67 KB DOC)

Table S3 Functions of High-Scoring Candidates for Translation on Demand (MIPS) (38 KB DOC)

Table S4 Functions of High-Scoring Candidates for Translation on Demand (SGD) (44 KB DOC)

Table S5 Proteins with Highest Concentration Changes under Stress Conditions in Comparison with the Corresponding mRNA Changes (55 KB DOC)

Table S6 Ratio of Protein Concentration Change versus TA Change for All 62 Proteins with Measured mRNA and Protein Concentrations under All Four Conditions (137 KB DOC)

## Abbreviations

CAI: codon adaptation index
GCN: gene copy number
MIPS: Munich Information Center for Protein Sequences
PC: protein change
PHD: protein half-life descriptors, PRR, proteins per mRNA
*ribden*: ribosome density
*ribocc*: ribosome occupancy
*ribocc* × *ribden*: the product of ribosome occupancy and ribosome density
*r_s_*: Spearman rank correlation coefficient
RSCU: relative synonymous codon usage
SGD: Saccharomyces Genome Database
TA: translational activity
TC: change of translation rate
tRNA–AI: tRNA adaptation index
