# Supplementary material for: Posttranscriptional Expression Regulation: What Determines Translation Rates?
Source: PLoS Comput Biol. 2007 Mar 23;3(3):e57. doi: 10.1371/journal.pcbi.0030057 (PMC1829480; doi:10.1371/journal.pcbi.0030057)
Supplement: Supplementary file 1 [file pcbi.0030057.sd001.doc]

**Protocol S1**

Saturation effects in the translation rate and calculation of protein half-lives

Fig. S1 shows that the type of kinetics suggested by equation (3) is independent of the experimental techniques employed.

**Figure S1: Determination of the optimal *K_m_* for TA3.**

TA3 is correlated to different protein datasets to find the optimal *K_m_*. The protein concentrations of Ghaemmaghami *et al.* (2004) were used as training set. The test set was obtained by merging the other three datasets (Gygi *et al*., 1999; Futcher *et al*., 1999; Liu *et al*., 2004). ‘All Data’ denotes the final protein concentration dataset (see Methods). The optimal K_m_ obtained for the test set also improves the TA3 correlation based on the other datasets.

Post-transcriptional expression regulation in different functional modules

Fig. S2 shows that functionally related genes behave similarly at different regulatory levels (Beyer *et al.*, 2004, Nie *et al.*, 2006). Again, a consistent improvement of the correlations can be observed when using *TA*3 instead of *TA*1 or *TA*2, suggesting that the kinetics assumed for *TA*3 is not specific for certain functional groups (Fig. S3 shows the corresponding average PHD). Some modules exhibit a stronger correlation between protein and mRNA concentrations than others, implying that production of the former proteins is mainly regulated at the transcriptional level (Beyer *et al.,* 2004). Most interestingly, there is a substantially improved correlation with respect to TA3 for the modules ‘protein activity regulation’, ‘interaction with environment’ and ‘signal transduction’. Many of these proteins may be subject to ‘translation on demand’ (Beyer *et al.*, 2004; Law *et al.*, 2006), i.e. their mRNA is held available and translation is turned on when triggered by the respective signal.

**Figure S2: Post-transcriptional regulation in different functional modules (MIPS classification).**

Spearman rank correlation coefficients (*r_s_*, all *p*<E-11 (exception: DV)) for mRNA and TA versus protein concentrations for all modules containing >10 ORFs under standard conditions. Modules are: MB=metabolism; EG=energy; CC=cell cycle and DNA processing; TC=transcription; PS=protein synthesis; PF=protein fate; PB=protein with binding function or cofactor requirement; AR=protein activity regulation; TP=cellular transport, transport facilitation and transport routes; ST=cellular communication /signal transduction mechanism; CR=cell rescue, defence and virulence; IE=interaction with the cellular environment; IS=interaction with environment; VP=transposable elements, viral and plasmid proteins; CF=cell fate; DV=development (*p*<E-4); BG=biogenesis of cellular components; DF=cell type differentiation; UC=unknown components.

**Figure S3: Relative protein half-lives in different functional modules.**
Median values are shown for each group of genes. Modules are the same as in Fig. S2.

The analysis of stress conditions confirms the general conclusions of the standard condition analysis: protein and mRNA changes are better correlated in the modules ‘metabolism’ and ‘energy’ than in modules related to signal transduction (ST, AR, IE; Fig. S4), which is in excellent agreement with recent experimental data (Kolkman *et al.* 2006). There is no significant correlation at all in the pheromone dataset (Fig. S4). However, this measurement does not depict a steady-state situation (measured already 30min after pheromone supply). Thus, protein concentrations may have changed much later in response to the mRNA changes.

**Figure S4: Correlation of relative changes upon exposure to stress conditions in different functional modules.**

Modules are the same as in Fig. S2. Correlations with <10 values are not shown.
