# Supplementary material for: Posttranscriptional Expression Regulation: What Determines Translation Rates?
Source: PLoS Comput Biol. 2007 Mar 23;3(3):e57. doi: 10.1371/journal.pcbi.0030057 (PMC1829480; doi:10.1371/journal.pcbi.0030057)
Supplement: Supplementary file 2 [file pcbi.0030057.st002.doc]

# Table S2: Correlation of measured and calculated protein half-lives

| **Protein** | **Std-Name** | **PHD_old_^1^** | **PHD_new_^2^** | **Measured^3^** | **Ref.** | **Δ_old_** | **Δ_new_** |
| --- | --- | --- | --- | --- | --- | --- | --- |
| FAR1 | YJL157c | 5 | 5 | 25 | (1) | 0.78 | 0.78 |
| P1beta | YDL130w | 3 | 89 | 15 | (2) | 0.78 | 4.93 |
| P2beta | YDR382w | 134 | 1193 | 300 | (2) | 0.55 | 2.98 |
| HMG2 | YLR450w | 121 | 27 | 55 | (3) | 1.19 | 0.51 |
| HMG1 | YML075c | 136 | 70 | 240 | (3) | 0.43 | 0.71 |
| RPL40A | YIL148w | 55 | 82 | 120 | (4) | 0.55 | 0.32 |
| CLN2 | YPL256c | 38 | 23 | 20 | (5) | 0.88 | 0.14 |
| CLB5 | YPR120c | 75 | 17 | 15 | (6) | 4.01 | 0.11 |
| CLB2 | YPR119w | 119 | 18 | 15 | (6) | 6.90 | 0.18 |
| CBF1 | YJR060w | 220 | 160 | 60 | (7) | 2.66 | 1.66 |
| MET4 | YNL103w | 359 | 58 | 30 | (7) | 10.96 | 0.94 |
| MET30 | YIL046w | 25 | 8 | 60 | (5) | 0.59 | 0.87 |
| MET31 | YPL038w | 28 | 19 | 30 | (7) | 0.06 | 0.38 |
| RFA1 | YAR007c | 198 | 74 | 35 | (8) | 4.66 | 1.13 |
| RAD51 | YER095w | 163 | 124 | 120 | (9) | 0.35 | 0.03 |
| RAD52 | YML032c | 135 | 34 | 15 | (9) | 7.99 | 1.28 |
| Gic2 | YDR309c | 42 | 24 | 30 | (5) | 0.40 | 0.21 |
| TAT2 | YOL020w | 44 | 18 | 90 | (10) | 0.51 | 0.80 |
| GRX5 | YPL059w | 142 | 109 | 240 | (11) | 0.41 | 0.55 |
| GLC7 | YER133w | 195 | 173 | 180 | (12) | 0.08 | 0.04 |
| PAP1 | YKR002w | 336 | 218 | 840 | (13) | 0.60 | 0.74 |
| STE2 | YFL026w | 36 | 66 | 72 | (14) | 0.50 | 0.09 |
| **Sum** |  |  |  |  |  | 46 | 19 |

1. PHDold = prot/(mRNA*ribocc*ribden) (cf. Tab. S1)

2. PHDnew = prot/TA3 (eq. 4)

3. Measured protein half-lives (min)

(1) Blondel et al. (2000) *EMBO J*. 19(22):6085-97

(2) Nusspaumer et al. (2000) *EMBO J*. 19(22):6075-84

(3) Hampton, Rine (1994) *J Cell Biol.* 125:299-312

(4) Hanna et al. (2003) *Mol Cell Biol*. 23(24):9251-61

(5) Blondel et al. (2000) *Genetics* 155(3):1033-44

(6) MIPS (value cited at http://mips.gsf.de without reference)

(7) Rouillon et al. (2000) *EMBO J*. 19(2):282-94

(8) Erdeniz, Rothstein (2000) *Mol Cell Biol*. 20(1):224-32.

(9) Asleson, Livingston 2003 *Genetics* 163(1):91-101

(10) Beck et al. (1999) *J Cell Biol*. 146(6):1227-38

(11) Belli et al. (2002) *J Biol Chem*. 277(40):37590-6

(12) Nigavekar et al. (2002) *Arch Biochem Biophys*. 404(1):71-9

(13) Mizrahi, Moore (2000) *Mol Cell Biol*. 20(8):2794-802

(14) Jenness, et al. (1997) *Mol Cell Biol*. 17(11):6236-45

In order to facilitate the comparison, PHDs were normalized to match the mean half-life of the measured proteins. The relative deviations between predicted and measured values (Δ_old_, Δ_new_) were computed as |*predicted-measured|* / *measured*. The Spearman rank correlation coefficient (*r_s_*) between the old PHDs and the measured values is 0.43 (*p* = 0.046), the corresponding *r_s_* for the new PHDs is 0.64 (*p* = 0.0013).
