# Supplementary material for: Posttranscriptional Expression Regulation: What Determines Translation Rates?
Source: PLoS Comput Biol. 2007 Mar 23;3(3):e57. doi: 10.1371/journal.pcbi.0030057 (PMC1829480; doi:10.1371/journal.pcbi.0030057)
Supplement: Supplementary file 3 [file pcbi.0030057.st003.doc]

**Table S3:** Functions of high scoring candidates for translation on demand (MIPS)

| **Category Name (MIPS) ^1^** | **p-value ^2^** | **N Category ^3^** | **N Overlapping ^4^** |
| --- | --- | --- | --- |
| *Overlap with Top 100* |  |  |  |
| mRNA synthesis ^5^ | 2.7E-06 | 570 | 24 |
| transcriptional control | 2.9E-06 | 492 | 22 |
| osmosensing | 8.3E-06 | 33 | 6 |
| cellular communication/signal transduction | 4.6E-05 | 234 | 13 |
| *Overlap with Top 500* |  |  |  |
| transcriptional control | 1.6E-12 | 492 | 82 |
| mRNA synthesis ^5^ | 1.8E-11 | 570 | 88 |
| cell cycle & DNA processing | 5.0E-11 | 994 | 129 |
| cell cycle | 1.1E-08 | 644 | 88 |
| mitotic cell cycle and cell cycle control | 6.7E-08 | 440 | 65 |
| transcription | 9.7E-08 | 1026 | 121 |
| chemoperception and response | 1.6E-07 | 265 | 45 |
| cellular sensing and response | 1.6E-07 | 283 | 47 |
| mitotic cell cycle | 3.4E-07 | 162 | 32 |
| cellular communication/signal transduction | 6.6E-07 | 234 | 40 |
| intracellular signaling | 4.2E-06 | 198 | 34 |
| transcriptional activator | 5.6E-06 | 41 | 13 |

1. Annotation based on MIPS. Only the most significant overlaps are shown.
2. Probability of chance overlap (assuming hypergeometric distribution).
3. Number of genes in the category.
4. Number of genes overlapping with top scoring candidates.
5. This category subsumes all transcription factors.
