# Supplementary material for: Posttranscriptional Expression Regulation: What Determines Translation Rates?
Source: PLoS Comput Biol. 2007 Mar 23;3(3):e57. doi: 10.1371/journal.pcbi.0030057 (PMC1829480; doi:10.1371/journal.pcbi.0030057)
Supplement: Supplementary file 4 [file pcbi.0030057.st004.doc]

**Table S4:** Functions of high scoring candidates for translation on demand (SGD)

| **Category Name (SGD) ^1^** | **p-value ^2^** | **N Category ^3^** | **N Overlapping ^4^** |
| --- | --- | --- | --- |
| *Overlap with Top 100* |  |  |  |
| transcription regulator activity | 3.2E-06 | 322 | 18 |
| regulation of biological process | 1.3E-05 | 544 | 23 |
| MAP kinase kinase activity | 1.6E-05 | 4 | 3 |
| RNA polymerase II transcription factor activity | 2.4E-05 | 122 | 10 |
| regulation of cellular physiological process | 8.5E-05 | 488 | 20 |
| *Overlap with Top 500* |  |  |  |
| regulation of biological process | 1.6E-13 | 544 | 94 |
| regulation of physiological process | 4.2E-13 | 535 | 92 |
| regulation of cellular physiological process | 7.1E-13 | 488 | 86 |
| transcription regulator activity | 3.4E-10 | 322 | 60 |
| cell organization and biogenesis | 1.1E-08 | 845 | 113 |
| regulation of metabolism | 1.6E-08 | 389 | 64 |
| regulation of cellular metabolism | 3.2E-08 | 335 | 57 |
| regulation of transcription | 5.2E-08 | 297 | 52 |
| regulation of transcription, DNA-dependent | 7.1E-08 | 283 | 50 |
| regulation of nucleobase, nucleoside, nucleotide and nucleic acid metabolism | 1.5E-07 | 315 | 53 |
| regulation of progression through cell cycle | 1.6E-07 | 133 | 30 |
| biological process | 1.0E-06 | 5670 | 485 |
| nucleus | 1.7E-06 | 1106 | 130 |
| organelle organization and biogenesis | 1.7E-06 | 692 | 90 |
| site of polarized growth | 2.1E-06 | 119 | 26 |
| RNA polymerase II transcription factor activity | 3.4E-06 | 122 | 26 |
| specific RNA polymerase II transcription factor activity | 3.8E-06 | 43 | 14 |

1. Annotation based on SGD (GO-categories). Only the most significant overlaps are shown.
2. Probability of chance overlap (assuming hypergeometric distribution).
3. Number of genes in the category.
4. Number of genes overlapping with top scoring candidates.
