# Supplementary material for: Posttranscriptional Expression Regulation: What Determines Translation Rates?
Source: PLoS Comput Biol. 2007 Mar 23;3(3):e57. doi: 10.1371/journal.pcbi.0030057 (PMC1829480; doi:10.1371/journal.pcbi.0030057)
Supplement: Supplementary file 5 [file pcbi.0030057.st005.doc]

**Table S5:** Proteins with highest concentration changes under stress conditions in comparison to the corresponding mRNA changes

| ***Gene ^1^*** | ***Gene Name*** | ***Function (MIPS)*** | ***mRNA Abnd.^2^*** | ***CAI*** | ***ribocc× ribdens ^3^*** | ***TA3 ^4^*** | ***Prot Abnd.^5^*** | ***PRR (ref) ^6^*** | ***PRR (stress) ^7^*** | ***ToD score^8^*** |
| --- | --- | --- | --- | --- | --- | --- | --- | --- | --- | --- |
| YLR422w | YLR422w | *unknown* | 0.69 | 0.12 | 0.04 | 0.04 | 238 | 345 | 2.06 | 0.29 |
| YNL172w | APC1 | cell cycle/protein fate | 0.71 | 0.12 | 0.08 | 0.06 | 178 | 251 | 21.0 | 0.39 |
| YOR227w | YOR227w | *unknown* | 0.52 | 0.15 | 0.08 | 0.06 | 195 | 374 | 3.53 | 0.44 |
| YOL055c | THI20 | transcription factor/phosphate utilization | 0.25 | 0.13 | 0.05 | 0.02 | 259 | 1036 | 2.03 | 0.57 |
| YMR176w | ECM5 | cell wall | 0.25 | 0.26 | 0.08 | 0.06 | 195 | 779 | 2.54 | 0.57 |
| YKL075c | YKL075c | *unknown* | 0.33 | 0.17 | 0.13 | 0.07 | 99 | 300 | 4.99 | 0.58 |
| YJL042w | MHP1 | cell wall/microtuble formation | 2.21 | 0.15 | 0.15 | 0.13 | 279 | 126 | 2.21 | 0.59 |
| YER132c | PMD1 | regulator of meiotic expression | 0.95 | 0.12 | 0.08 | 0.07 | 815 | 858 | 4.01 | 0.61 |
| YBR007c | DSF2 | *unknown* | 0.46 | 0.12 | 0.11 | 0.05 | 377 | 819 | 42.6 | 0.70 |
| YER005w | YND1 | metabolism, protein fate | 1.27 | 0.12 | 0.16 | 0.09 | 450 | 355 | 3.76 | 0.70 |
| YLL021w | SPA2 | intracellular signalling/cell communication | 0.93 | 0.15 | 0.09 | 0.09 | 1191 | 1281 | 11.7 | 0.83 |
| YDR356w | SPC110 | cell cycle/spindle pole component | 0.18 | 0.15 | 0.06 | 0.02 | 279 | 1552 | 8.59 | 0.84 |
| YPR124w | CTR1 | cellular transport/interact. wt. environment | 4.23 | 0.12 | 0.22 | 0.11 | 1184 | 280 | 2.45 | 0.89 |
| YLR352w | YLR352w | *unknown* | 0.15 | 0.17 | 0.18 | 0.05 | 125 | 830 | 43.6 | 0.96 |
| YOR231w | MKK1 | cell cycle/cell wall/differentiation | 0.73 | 0.11 | 0.12 | 0.06 | 1040 | 1425 | 2.17 | 0.97 |
| YJR024c | YJR024c | *unknown* | 3.69 | 0.12 | 0.25 | 0.11 | 952 | 258 | 2.31 | 0.98 |
| YLR167w | RPS31 | ubiquitin & ribosome subunit | 126 | 0.81 | 0.25 | 0.81 | 32368 | 256 | 2.18 | 0.98 |
| YBR133c | HSL7 | cell cycle/budding | 0.94 | 0.13 | 0.14 | 0.09 | 1184 | 1259 | 2.29 | 0.99 |
| YLR342w | FKS1 | metabolism/energy/cell wall | 7.89 | 0.27 | 0.11 | 0.26 | 11981 | 1519 | 3.27 | 1.00 |
| YOR034c | AKR2 | endocytosis/cell trafficking | 0.7 | 0.13 | 0.16 | 0.08 | 815 | 1164 | 10.0 | 1.01 |

1. High ranking translation on demand (ToD) candidates (top 500) with PRR changes > 2 under stress.
2. mRNA concentration under standard conditions
3. Product of ribosome occupancy and ribosome density under standard conditions
4. Transcriptional activity assuming Michaelis-Menten kinetics
5. Protein concentration under standard conditions
6. Ratio of protein and mRNA concentrations under standard conditions
7. Maximum ratio of protein and mRNA concentration changes under one of the four tested stress conditions
8. Translation on demand score (weighted sum of *ribocc*×*ribdens* and the PRR) under standard condition
