# Supplementary material for: Posttranscriptional Expression Regulation: What Determines Translation Rates?
Source: PLoS Comput Biol. 2007 Mar 23;3(3):e57. doi: 10.1371/journal.pcbi.0030057 (PMC1829480; doi:10.1371/journal.pcbi.0030057)
Supplement: Supplementary file 6 [file pcbi.0030057.st006.doc]

**Table S6:** Ratio of protein concentration change versus TA change for all 62 proteins with measured mRNA and protein concentrations under all four conditions.^1^

|  |  |  | before accounting for saturation | | |  | after accounting for saturation | | |  |
| --- | --- | --- | --- | --- | --- | --- | --- | --- | --- | --- |
| **Gene** |  | **Function** | **+/- galact.^2^** | **+/-ethan^3^** | **min med.^4^** | **pherom.^5^** | **+/- galact.** | **+/- ethan.** | **min med.** | **pherom.** |
| YOR375c | GDH1 | amino acid metabolism, energy | 0.23 | 0.35 | 1.17 | **1.37** | 0.53 | 1.00 | **7.47** | 1.03 |
| YNL134c | YNL134c | putative alcohol dehydrogenase | 0.73 | 1.82 | **3.05** | 3.01 | 0.94 | 0.54 | **6.22** | 1.10 |
| YPL028w | ERG10 | ergosterol biosynthesis | **9.71** | 1.32 | 0.86 | 1.85 | **6.68** | 1.15 | 0.68 | 1.18 |
| YER062c | HOR2 | glycerol biosynthesis, stress response | 0.09 | 0.60 | 2.37 | **5.11** | 1.33 | 0.23 | **5.07** | 1.11 |
| YPR191w | QCR2 | protein fate, energy | 0.70 | 0.42 | **2.39** | 0.49 | 0.60 | 1.08 | **3.08** | 0.73 |
| YGL137w | SEC27 | golgi-to-ER transport | 0.90 | 0.55 | **3.20** | 1.02 | 0.89 | 0.57 | **3.01** | 0.90 |
| YKL148c | SDH1 | aerobic respiration, electron transport | **5.27** | 0.30 | 3.85 | 1.54 | 0.87 | 0.64 | **3.24** | 1.14 |
| YNL055c | POR1 | aerobic respiration, ion channel | 1.47 | 0.68 | **2.23** | 1.39 | 0.79 | 0.66 | **2.65** | 0.94 |
| YML085c | TUB1 | nuclear migration (tubulin) | 1.52 | 0.21 | 2.99 | **2.98** | 0.89 | 0.34 | **3.36** | 1.32 |
| YNR050c | LYS9 | amino acid metabolism | 0.74 | 0.21 | 1.09 | **1.63** | 1.25 | 0.96 | **3.08** | 1.16 |
| YJR016c | ILV3 | amino acid metabolism | 0.68 | 0.39 | **1.58** | 1.53 | 1.00 | 0.50 | **2.57** | 1.11 |
| YGL026c | TRP5 | amino acid metabolism | 0.97 | 0.27 | 1.35 | **1.96** | 0.94 | 0.31 | **2.45** | 1.10 |
| YGL245w | GUS1 | tRNA metabolism | 1.21 | 0.43 | 2.12 | **2.81** | 0.89 | 0.62 | **1.95** | 0.77 |
| YDL126c | CDC48 | proteasomal degradation, cell cycle control | 0.88 | **4.57** | 1.52 | 2.57 | 0.96 | **2.64** | 1.22 | 1.15 |
| YER043c | SAH1 | amino acid metabolism | 0.53 | 0.46 | **1.80** | 1.39 | 0.82 | 0.71 | **1.96** | 0.95 |
| YKL127w | PGM1 | sugar metabolism, energy | 0.83 | 0.71 | 0.69 | **0.98** | 0.52 | 0.76 | 0.55 | **1.42** |
| YLR058c | SHM2 | amino acid metabolism | 0.07 | 0.20 | 0.72 | **1.59** | 1.26 | 0.48 | **1.90** | 0.61 |
| YMR297w | PRC1 | protein degradation | **2.24** | 1.10 | 1.87 | 1.38 | 0.87 | 1.17 | **2.22** | 1.01 |
| YOR142w | LSC1 | TCA cycle | 0.62 | 0.74 | 1.58 | **1.77** | 0.53 | 0.72 | **2.21** | 1.47 |
| YDL185w | TFP1 | cellular transport, DNA integration | 1.33 | 1.02 | 1.93 | **2.58** | 1.11 | 0.85 | **2.16** | 1.06 |
| YDR071c | PAA1 | amino acid metabolism, may be involved in transcription | 0.35 | 0.63 | **1.57** | 0.42 | 0.86 | 0.68 | **1.49** | 0.62 |
| YLR180w | SAM1 | amino acid metabolism | 0.17 | 0.38 | 0.14 | **1.69** | **1.52** | 0.49 | 0.32 | 1.09 |
| YDR037w | KRS1 | tRNA metabolism | 0.74 | 0.50 | **1.04** | 0.77 | **1.53** | 0.85 | 0.72 | 0.84 |
| YDR158w | HOM2 | amino acid metabolism | 1.06 | **1.45** | 1.36 | 1.29 | 1.26 | 1.12 | **2.08** | 1.00 |
| YLR216c | CPR6 | transcription factor, protein folding | **1.71** | 0.45 | 1.04 | 0.67 | **1.24** | 0.52 | 0.66 | 0.84 |
| YFL045c | SEC53 | sugar metabolism, protein transport | 0.80 | 0.74 | 0.79 | **1.12** | **1.26** | 0.76 | 0.73 | 0.76 |

Table S6 cntd.

|  |  |  | before accounting for saturation | | |  | after accounting for saturation | | |  |
| --- | --- | --- | --- | --- | --- | --- | --- | --- | --- | --- |
| **Gene** |  | **Function** | **+/- galact.** | **+/- ethan.** | **min med.** | **pherom.** | **+/- galact.** | **+/- ethan.** | **min med.** | **pherom.** |
| YBR031w | RPL4A | ribosomal protein | **1.21** | 0.46 | 0.86 | 0.97 | **1.03** | 0.54 | 0.60 | 0.64 |
| YLR259c | HSP60 | stress response, cellular transport | 1.64 | 0.41 | 1.04 | **2.15** | 0.92 | 0.71 | 0.61 | **1.34** |
| YBL087c | RPL23A | ribosomal protein | 0.27 | 0.36 | 0.89 | **1.12** | **1.34** | 0.59 | 0.72 | 0.99 |
| YLR075w | RPL10 | ribosomal protein, translation initiation | 0.24 | 0.22 | 0.52 | **1.06** | **1.06** | 0.26 | 0.38 | 1.04 |
| YPR145w | ASN1 | amino acid metabolism | 0.42 | 0.49 | 0.29 | **1.31** | 0.90 | 0.83 | 0.17 | **1.29** |
| YLR175w | CBF5 | nucleotide metabolism, rRNA processing | 0.16 | 0.32 | 0.96 | **1.33** | 0.99 | 0.35 | 0.67 | **1.19** |
| YBR048w | RPS11B | ribosomal protein | 0.36 | 0.20 | **1.12** | 1.02 | **1.29** | 0.48 | 0.73 | 1.08 |
| YGR234w | YHB1 | stress response | 0.23 | 0.69 | **1.29** | 1.22 | 0.59 | 0.81 | 0.11 | **1.00** |
| YGL202w | ARO8 | amino acid metabolism | **1.77** | 0.93 | 1.75 | 1.47 | **2.38** | 1.17 | 2.27 | 1.05 |
| YLR304c | ACO1 | amino acid & sugar metabolism | **3.15** | 0.17 | 1.14 | 1.82 | 0.80 | 0.77 | **1.31** | 1.11 |
| YDL055c | PSA1 | sugar metabolism | 0.69 | 0.45 | 1.38 | **1.82** | 1.10 | 0.71 | **1.47** | 1.08 |
| YPL061w | ALD6 | C-2 compound and organic acid anabolism, energy | **69.18** | 0.45 | 0.69 | 1.05 | **1.42** | 1.14 | 0.92 | 0.96 |
| YML008c | ERG6 | isoprenoid biosynthesis | 0.76 | 0.60 | **1.15** | 0.82 | **1.17** | 0.83 | 0.76 | 0.92 |
| YLR197w | SIK1 | rRNA processing | 0.11 | 0.43 | **0.99** | 0.84 | **0.99** | 0.49 | 0.66 | 0.84 |
| YAL038w | CDC19 | sugar & nucleotide metabolism | 0.66 | 1.66 | 1.46 | **2.17** | 0.83 | 0.37 | 0.99 | **1.20** |
| YLR153c | ACS2 | glucose metabolism | 1.52 | 0.29 | 1.07 | **2.60** | 1.12 | 0.99 | 0.89 | **1.36** |
| YBL072c | RPS8A | ribosomal protein | 0.23 | 0.22 | 0.68 | **0.81** | **0.92** | 0.42 | 0.57 | 0.86 |
| YLR109w | AHP1 | stress response, detoxification | **2.64** | 0.27 | 1.33 | 1.48 | **1.51** | 0.22 | 1.39 | 0.99 |
| YDR418w | RPL12B | ribosomal protein | 0.34 | 0.22 | 0.94 | **1.39** | **1.11** | 0.76 | 0.71 | 0.99 |
| YFL039c | ACT1 | actin | 1.52 | 0.37 | 1.22 | **2.28** | 0.83 | 0.47 | 1.01 | **1.16** |
| YBR143c | SUP45 | translation termination | 0.48 | 0.28 | 0.97 | **1.80** | **1.15** | 0.41 | 0.77 | 1.05 |
| YJL026w | RNR2 | DNA synthesis | **1.67** | 0.56 | 0.61 | 1.38 | 1.01 | 0.77 | 0.62 | **1.10** |
| YBR025c | YBR025c | unkown | 0.77 | 0.72 | 0.98 | **1.51** | 1.13 | 0.81 | 0.66 | **1.20** |
| YKL182w | FAS1 | amino acid & fatty acid metabolism | 1.22 | 0.81 | 0.92 | **2.96** | 1.14 | 0.63 | 1.18 | **1.42** |
| YLR355c | ILV5 | amino acid metabolism | 0.27 | 0.16 | 0.54 | **1.53** | 0.73 | 0.54 | 0.94 | **1.03** |
| YER178w | PDA1 | energy | **2.29** | 0.51 | 1.08 | 1.32 | 0.89 | 0.72 | 0.81 | **1.03** |
| YLR293c | GSP1 | rRNA processing, nuclear transport | 0.49 | 0.52 | **0.93** | 0.41 | **0.93** | 0.81 | 0.74 | 0.68 |
| YKL060c | FBA1 | sugar metabolism, energy | 0.97 | 0.68 | **1.64** | 1.62 | 0.98 | 0.21 | **1.23** | 1.14 |

Table S6 cntd.

|  |  |  | before accounting for saturation | | |  | after accounting for saturation | | |  |
| --- | --- | --- | --- | --- | --- | --- | --- | --- | --- | --- |
| **Gene** |  | **Function** | **+/- galact.** | **+/- ethan.** | **min med.** | **pherom.** | **+/- galact.** | **+/- ethan.** | **min med.** | **pherom.** |
| YNR016c | ACC1 | fatty acid biosynthesis, nuclear transport | 1.05 | **1.38** | 1.26 | 3.04 | 0.92 | 1.32 | **1.39** | 1.09 |
| YGL135w | RPL1B | ribosomal protein | 0.42 | 0.49 | 0.39 | **1.25** | **1.11** | 0.85 | 0.32 | 1.08 |
| YHR068w | DYS1 | amino acid metabolism | 0.32 | 0.68 | **1.13** | 1.11 | 0.97 | 0.62 | 0.89 | **1.06** |
| YKL035w | UGP1 | sugar & phosphate metabolism, protein modification | **3.13** | 1.17 | 1.72 | 1.40 | 1.31 | 1.17 | **1.41** | 1.11 |
| YBR121c | GRS1 | tRNA synthesis, transcription factor | 0.56 | 0.34 | 0.88 | **4.76** | 0.90 | 0.45 | 0.79 | **0.96** |
| YNL301c | RPL18B | ribosomal protein | 0.35 | 0.15 | **1.40** | 1.16 | 0.82 | 0.32 | 1.04 | **1.05** |
| YBL076c | ILS1 | tRNA metabolism | 0.31 | 0.59 | 1.21 | **2.30** | 1.20 | 0.53 | 1.22 | **1.28** |
| YML028w | TSA1 | stress response, detoxification | **1.74** | 0.63 | 1.37 | 1.42 | 1.06 | 0.85 | 1.07 | **1.11** |

1. Maximum ratio is highlighted for each protein.
2. Ratio in response to +/- galactose conditions (Ideker *et al*. 2001).
3. Ratio in response to +/- ethanol conditions (Griffin *et al*. 2002).
4. Ratio in response to amino acid starvation (minimal media) (Washburn *et al*. 2001).
5. Ratio in response to pheromone (MacKay *et al*. 2004).
